# Effects of Cutting Edge Microgeometry on Residual Stress in Orthogonal Cutting of Inconel 718 by FEM

**DOI:** 10.3390/ma11061015

**Published:** 2018-06-14

**Authors:** Qi Shen, Zhanqiang Liu, Yang Hua, Jinfu Zhao, Woyun Lv, Aziz Ul Hassan Mohsan

**Affiliations:** 1 School of Mechanical Engineering, Shandong University, Jinan 250061, China; 201612804@mail.sdu.edu.cn (Q.S.); sduhuayang@gmail.com (Y.H.); sduzhaojinfu@gmail.com (J.Z.); sdulvwoyun@gmail.com (W.L.); hassansdu@yahoo.com (A.U.H.M.); 2 Key Laboratory of High Efficiency and Clean Mechanical Manufacture of MOE/Key National Demonstration Center for Experimental Mechanical Engineering Education, Jinan 250061, China

**Keywords:** cutting edge microgeometry, residual stress, finite element model, cutting edge preparation, Inconel 718

## Abstract

Service performance of components such as fatigue life are dramatically influenced by the machined surface and subsurface residual stresses. This paper aims at achieving a better understanding of the influence of cutting edge microgeometry on machined surface residual stresses during orthogonal dry cutting of Inconel 718. Numerical and experimental investigations have been conducted in this research. The cutting edge microgeometry factors of average cutting edge radius $\bar{S}$, form-factor *K*, and chamfer were investigated. An increasing trend for the magnitudes of both tensile and compressive residual stresses was observed by using larger $\bar{S}$ or introducing a chamfer on the cutting edges. The ploughing depth has been predicted based on the stagnation zone. The increase of ploughing depth means that more material was ironed on the workpiece subsurface, which resulted in an increase in the compressive residual stress. The thermal loads were leading factors that affected the surface tensile residual stress. For the unsymmetrical honed cutting edge with *K* = 2, the friction between tool and workpiece and tensile residual stress tended to be high, while for the unsymmetrical honed cutting edge with *K* = 0.5, the high ploughing depth led to a higher compressive residual stress. This paper provides guidance for regulating machine-induced residual stress by edge preparation.

## 1. Introduction

More research attention has been paid to high performance and high process reliability in production due to the increasing demand for difficult-to-machine materials such as nickel alloys in the aerospace industry [1,2]. Aero-engine components such as turbine disks and shafts are subjected to huge and complex alternating loads during service conditions. Their service performance depends on their surface low cycle fatigue life, which is mainly influenced by the machine-induced surface residual stresses [3]. Specifically, the residual stresses impose an additional stress state on the surface of machined components during operation. The tensile residual stress tends to engender crack initiation and propagation, which then contributes to the diminution of fatigue life, whereas the compressive residual stress is conducive to fatigue life by suppressing crack propagation. Therefore, regulating the final stress condition during the cutting process is of paramount importance.

To improve the state of residual stress, a number of attempts have been made concerning the cutting parameters, cutting conditions, and macroscopic geometric parameters of cutting tools [4,5,6,7,8,9]. However, when these conditions are determined, there is still a consensus that the state of residual stress can be further improved. Recent research has proved that the cutting edge microgeometry has a direct influence on the deformation zone, cutting temperature distribution, and ploughing force; therefore, the cutting edge microgeometry plays a crucial role in the formation of residual stress [10,11,12].

The commonly applied edge preparation includes honed cutting edge (symmetrical honed edge and waterfall shape edge) and chamfered cutting edge. Ozel and Ulutan [13] investigated the influence of honed radius combined with the coating condition by three-dimensional FEM (finite element method). Their findings revealed that larger honed radii have the tendency to intensify both the magnitude of tensile residual stress and compressive residual stress. The influence of chamfered edge on residual stress was reported by Varela et al. [14]. Their findings revealed that the chamfered edge facilitates the compressive residual stress during hard turning. The formation of residual stress was also found to be closely related to the thermal properties of workpiece materials. Nasr et al. [15] observed that the thickness of tensile stress layer was not affected by applying a larger honed radius. This may be caused by the low thermal conductivity of the workpiece material, which restricted the effects of temperature rise. Attempts have also been made to figure out the causes behind these results. Ventura et al. [16] explained the larger and deeper compressive residual stress introduced by waterfull honed edge with smaller form-factor K through more intense friction in the deformation zone. The contact length between edge geometries and the material for honed edges with different K were investigated. Results indicated that the magnitude and the depth both increased with the contact length, which confirmed his explanation. The increase of tensile residual stress for larger honed radius has been explained by Nasr et al. [15]. The tensile residual stresses by-depth distribution was compared with workpiece temperature by-depth distribution for variate honed edge radii. High linear correlation was found in both parameters, which demonstrated that the increased thermal loads caused by tool/workpiece friction play a dominant role in increasing the tensile residual stress. Schulze et al. [17] investigated the scale effect of cutting edge radius on residual stress profiles in micro-cutting. Their findings revealed that the larger honed radius caused a deeper penetration of tensile residual stress due to the deeper plastic deformation. However, the explanation was given empirically and no concrete proof was applied through FE analysis.

Although numerous studies have been conducted focusing on the influences of cutting edge microgeometry on machined surface residual stress, there is still a misunderstanding about the influence of unsymmetrical honed cutting edge on machined surface residual stress. Understanding the causes of machined surface residual stress will help us better predict and control the states of residual stress during processing. The formation of residual stress is subjected to the coupled thermo-mechanical phenomenon during the cutting process. FEM can help give a deep insight into the cutting progress through the simulation of residual stress formation and distribution on machined surface [17,18]. However, few studies have attempted to further investigate the influence of microgeometry on formation mechanism of residual stress by FE analysis.

Thus, the following research aims to investigate the influences of cutting edge microgeometry on residual stress in orthogonal turning of Inconel 718 and try to provide physical explanations based on the cutting temperature by-depth distribution and the phenomenon of stagnation zone. Considering the variation of cutting edge microgeometry parameters is severely limited to the complexity of cutting edge preparation, a hybrid method of FE and cutting experiments is adopted. Unsymmetrical honed cutting edges are investigated based on the form–factor characterization method. The edge preparation through micro-blasting and the subsequent microgeometry measurements by Laser Scanning Confocal Microscopy (LSCM) are shown in this paper. To verify the reliability of FE model, the residual stresses of machined surface layer over a range of depth were measured by X-ray diffraction to compare with the simulation results.

## 2. Cutting Edge Characterization and Edge Preparation

**Figure 1:**
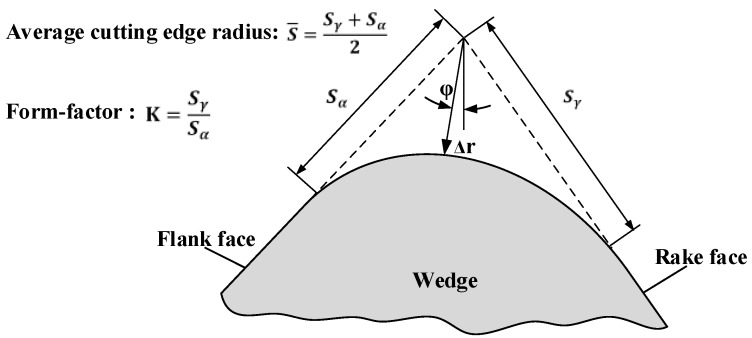
Cutting edge microgeometry characterized by *K*-factor.

For an unsymmetrical cutting edge, it is difficult to adjust the cutting edge profiles with a suitable circle. Characterization ambiguity and errors are inevitable with a honed edge radius *r_β_* in this case. Only the parameters of cutting edge are characterized accurately; its impact on cutting process can be reasonably predicted. The form-factor method established by Denkena et al. [19] is more appropriate for describing the honed cutting edge microgeometry. Four basic parameters used to measure the shape of the cutting edge are shown in Figure 1. *S_γ_*, *S_α_* are the distances from the separation point of the honed cutting edge to the tool tip of an ideal sharp cutting edge at the rake face and flank face, respectively. Average cutting edge rounding $\bar{S}$ is used to describe the dimension of honed rounding. Form-factor *K* (kappa) is introduced to indicate the offset level of the honed rounding to the flank face or the rake face. Profile flattening ∆*r* and apex angle *φ*, which are measured by the shortest distance and the shift between ideal sharp cutting edge tip and the actual shape of rounding, respectively, are used to characterize the tools’ bluntness.

**Figure 2:**
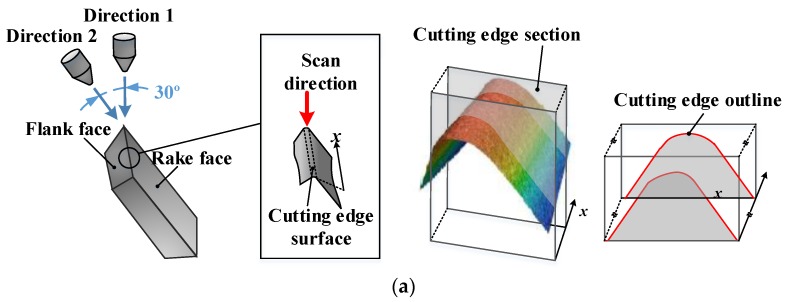

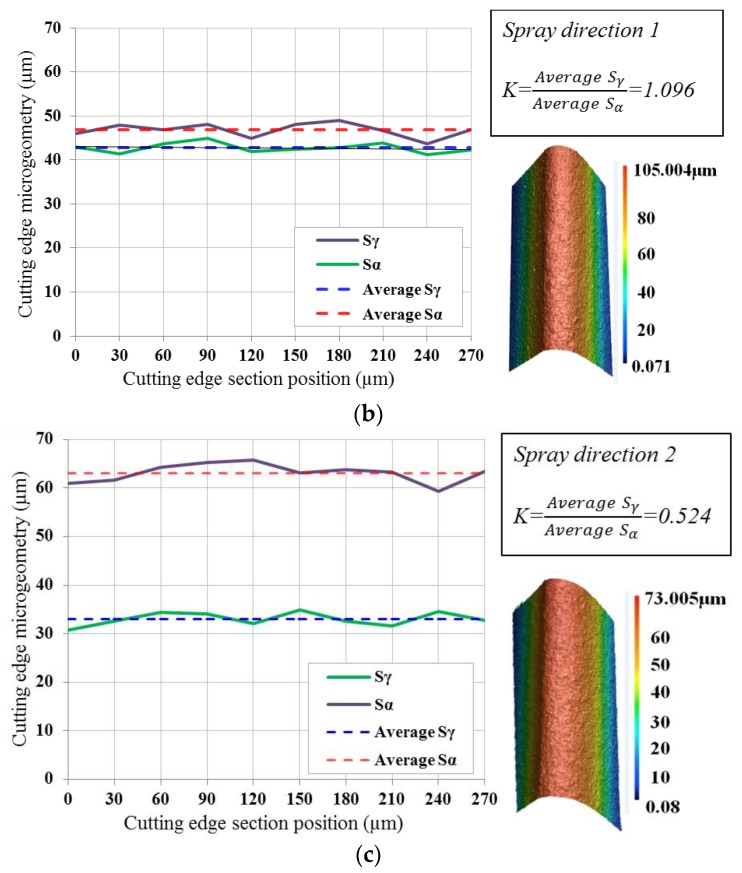
(**a**) Schematic of micro-blasting and the LSCM measurements of cutting edge microgeometry. (**b**) Cutting edge microgeometry measurements of spray direction 1 and (**c**) spray direction 2.

The wet micro-blasting experiments were carried out on a customized reciprocating type wet micro-blasting machine called SY-WF4W during edge preparation procedure. SECO tools LCGF160604-0600-GS with an initial average cutting edge radius $\bar{S}$ of about 10.5 μm, a wedge angle of 55° were adopted. Nickel-based superalloy is a typically difficult-to-machine material. The high shear strength, low thermal conductivity, and high hardness of its strengthening phases γ″ cause high cutting force and high cutting temperature during machining. Considering the problems above, non-coated tools with ultra-fine grain carbide matrix of 890 type from SECO Tools, which show excellent toughness and high hardness, were chosen. The abrasive particles were Al_2_O_3_ with an average size of 220 #. The injection pressure was set to 0.35 MPa. The blast gun sprayed from two different positions at an interval of 30° to process two different *K* of cutting edge microgeometries, as shown in Figure 2a. Laser scanning confocal microscopy (VK200, KEYENCE, Osaka, Japan) was used to measure the parameters of cutting edge microgeometry. Each cutting edge was measured 10 times repeatedly in a two-dimensional profile and an average value of *S_γ_* and *S_α_* were calculated. Two different values of *K* 0.524 and 1.096 were achieved, as shown in Figure 2b,c.

## 3. Orthogonal Experiments and Measurements of Residual Stress

The orthogonal turning experiments with Inconel 718 were conducted on a CNC lathe center (PUMA 200, DAEWOO, Changwon, Korea). Inconel 718 bars with an outline diameter of 70 mm and an average thickness of 3.5 mm were adopted. The micro-blasted cutting tools were used throughout the experimentation. The tool holder of SECO CFIR2525M06JET (SECO tools, Fagersta, Sweden) was adopted. After installation, the tools had a rake angle of 28°. The cutting speed was set to 29 m/min and the depth of cut was 0.15 mm.

**Figure 3:**
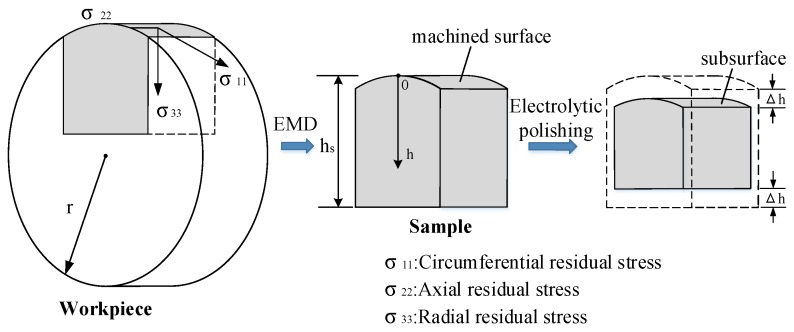
Measurements of residual stress in Inconel 718 sub-surface.

As shown in Figure 3, the machined surface samples were peeled off from the Inconel 718 plates by electrical discharge machining (EDM) after orthogonal cutting. The height of the samples *h_s_* was 15 mm and the width was 20 mm. Electrolytic polishing method was adopted to remove the surface material. After that, each surface of the samples was etched to a certain depth of *∆h*. By this method, the subsurface emerged for the measurements of residual stress. The parameters of the electrolytic polishing are shown in Table 1. EDM and electrolytic polishing are chemical processing methods that have little influence on the residual stress.

**Figure 4:**
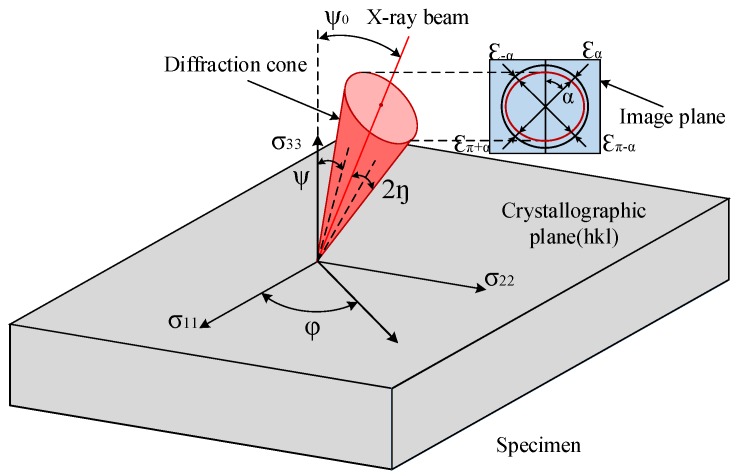
X-ray diffraction (XRD) schematic diagram.

A Pulstec μ-X360 X-ray diffraction apparatus (Pulstec, Hamamatsu, Japan) was used to measure the machined surface layer residual stresses. The X-ray diffractometer (XRD) technique is based on the measurement of the crystallographic lattice deformation. The cos*α* method, also called the single exposure method, was used to calculate the residual stress [20]. As shown in Figure 4, after diffraction in the atomic planes of crystal structure (also called crystallographic plane *hkl*), the diffraction cone of a single incident X-ray beam is formed. The diffraction cone is captured via a 2D detector in the image plane. The Debye ring is a regular circle for unstressed sample, while the Debye ring will be deformed for a stressed sample. The stress is calculated by comparing the amount of deformation between the Debye ring before and after stress. The lattice strain ${\bar{\varepsilon }}_{\alpha }^{\left(hkl\right)}$ is calculated as follows:

(1) $${\bar{\varepsilon }}_{\alpha }^{\left(hkl\right)}=\frac{1}{2}\left[\left(\varepsilon_{\alpha }^{\left(hkl\right)}-\varepsilon_{\pi +\alpha }^{\left(hkl\right)}\right)+\left(\varepsilon_{-\alpha }^{\left(hkl\right)}-\varepsilon_{\pi -\alpha }^{\left(hkl\right)}\right)\right],$$

where $\varepsilon_{\alpha }^{\left(hkl\right)}$, $\varepsilon_{\pi +\alpha }^{\left(hkl\right)}$, $\varepsilon_{-\alpha }^{\left(hkl\right)}$, $\varepsilon_{\pi -\alpha }^{\left(hkl\right)}$ are the strain of four points at an interval of 90° on the Debye ring. When α increases from 0 to 90°, each point on the Debye ring is contained. The values of ${\bar{\varepsilon }}_{\alpha }^{\left(hkl\right)}$ show a linear relationship with cos*α*. The stress calculated by cos*α* method is expressed as in Equation (2):

(2) $$\sigma_{\varphi }=\frac{E^{\left(hkl\right)}}{1+v^{\left(hkl\right)}}\frac{1}{\sin2\eta \sin2\psi_{0}}\frac{\partial {\bar{\varepsilon }}_{\alpha }^{\left(hkl\right)}}{\partial \cos\alpha }=\frac{1}{1/2S_{}^{\left(hkl\right)}}\frac{1}{\sin2\eta \sin2\psi_{0}}\frac{\partial {\bar{\varepsilon }}_{\alpha }^{\left(hkl\right)}}{\partial \cos\alpha }$$

(3) $$S^{\left(hkl\right)}=\frac{2(1+v^{\left(hkl\right)})}{E^{\left(hkl\right)}},$$

where 2*η* is the Debye ring semi-angle, *Ψ*_0_ is the constant tilt angle of X-ray beam. The elastic constant *S* in crystallographic plane (*hkl*) determined by Poisson’ ratio *v^(hkl)^* and elastic modulus *E^(hkl)^* of the specimen material is expressed as Equation (3).

X-ray tube current and voltage are 1 mA and 30 kV, respectively. X-ray incidence angle is 30°. Diffraction lattice angle 2*η* is 29.124°. The value of Poisson’s ratio *v^(hkl)^* and elastic modulus *E^(hkl)^* of Inconel 718 are 0.305 and 214.580 GPa, respectively. The elastic constant *S^(hkl)^* for the crystallographic plane is 1.216 × 10^−5^ MPa^−1^ by calculation.

## 4. Numerical Modeling

### 4.1. Geometry Modeling and Mesh Controlling

**Figure 5:**
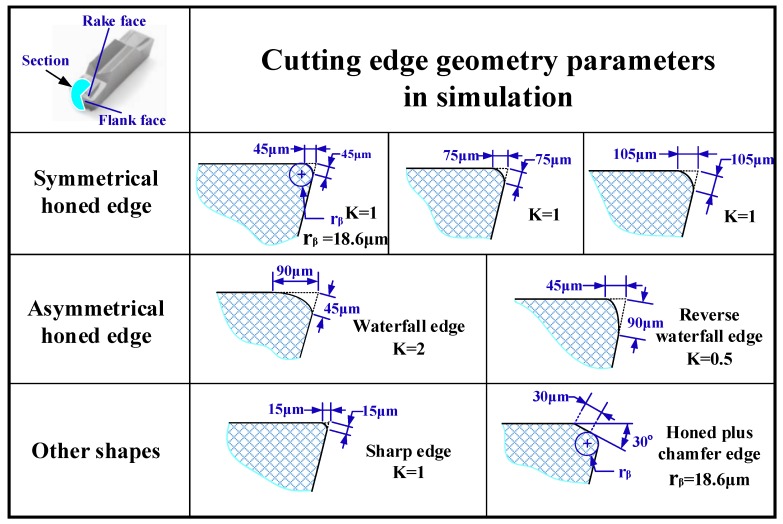
Cutting tool microgeometry parameters in simulations.

**Figure 6:**
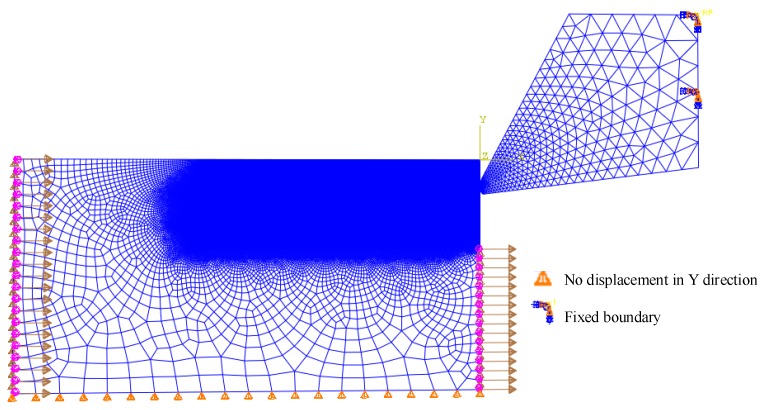
The boundary conditions and element configuration of the FE models in the initial position.

A two-dimensional plane strain FE model based on ABAQUS/Explicit was built to simulate the orthogonal dry turning of Inconel 718 superalloy with continuous chips. The symmetrical and unsymmetrical cutting edge models based on the form-factor method in the FE models are illustrated in Figure 5. The symmetrical cutting edge with $\bar{S}$ of 15 µm was used to represent the sharp edge. Moreover, a honed plus chamfer cutting edge was built to investigate the effect of chamfer on residual stress. In later discussions, the symmetrical cutting edges with $\bar{S}$ of 15 µm, 45 µm, 75 µm, 105 µm, the unsymmetrical cutting edge with *K* of 0.5, 2 and the honed plus chamfer cutting edge will be referred to as S15, S45(K1), S75, S105, K0.5, K2, Chamfer, respectively. The arbitrary Lagrangian–Eulerian (ALE) method was applied in the model. The ALE formulation combines the Lagrangian and Eulerian formulation during the re-meshing procedure to accommodate large deformation calculations. Two-dimensional triangle reduction integration element CPE3T was used in tool model. Considering the huge deformation of the workpiece, a relatively stable quadrilateral reduction integration element, CPE4RT, was used. The whole model was set as plane strain thermally coupled element, linear displacement, and temperature. To improve the simulation accuracy and shorten the simulation time, the mesh in the part of tool tip and the machined part of workpiece were locally refined (see Figure 6). In the final mesh models, about 140,000 quad elements were produced for the workpiece model and 900 tri elements were generated in the cutting tool model.

### 4.2. Initial Conditions

The implementation of boundary conditions is also shown in Figure 6. The cutting speed has to be applied to the workpiece when ALE is applied [21]. The cutting tool was fixed and the workpiece moved in the opposite direction to the cutting direction. The freedom of workpiece in Y direction was restricted. The initial temperature is set to be 20 °C (room temperature).

The interactions can be divided into three zones with different tribological properties: sticking zone, adhesion zone, and sliding zone. The sticking zone is distributed at the honed circle with high pressure, while the sliding zone is located between the sticking zone and the stagnation point in a low-pressure sliding friction state. To model the different friction zones, the Coulomb friction law, which has been widely adopted in FE simulation investigations of metal cutting [15,17,22], was used in the current work. The frictional shear stress *τ_f_* can be calculated by Equation (4):

(4) $$\tau_{f}=\{\begin{array}{l} \tau \;\;,\;\;\;\;\;\;\;\tau =\mu \sigma <\tau_{c}\;\;\;(\mathrm{Sticking}\;\;\mathrm{zone})\\ \tau_{c}\;\;,\;\;\;\;\;\;\;\tau =\mu \sigma >\tau_{c}\;\;\;(\mathrm{Sliding}\;\;\mathrm{zone}) \end{array},$$

where *μ* is the friction coefficient, *σ* is the normal stress, *τ* is the shear stress in tool/chip interface, and *τ_c_* is the limited shear stress. The friction coefficient *μ* was set to 0.3.

For the semi-empirical Johnson–Cook constitutive model, which reacts the thermal viscoplastic deformation behavior of material at high levels of strain, the strain rate was commonly chosen in the FE model of metal cutting [7,15,17,22]. The constitutive law is expressed with Equation (5). *A*, *B*, *C*, *m*, *n* are the yield stress, hardening modulus, strain rate dependency coefficient, thermal softening coefficient, and strain hardening coefficient, respectively; *T_m_* is the melting temperature of the material, *T_t_* is room temperature, *T* is the bulk temperature of workpiece, *ε* is equivalent strain rate, *ε*_0_ is the reference strain rate, $\bar{\varepsilon }$ is the equivalent strain, and *σ* is the equivalent stress. The Johnson–Cook constitutive parameters are specified in Table 2. The physical and thermomechanical properties of the tool and the workpiece material are presented in Table 3.

(5) $$\sigma =\left(A+B{\bar{\varepsilon }}^{n}\right)\left[1+C\ln\left(\frac{\varepsilon }{\varepsilon_{0}}\right)\right]\left[1-{\left(\frac{T-T_{r}}{T_{m}-T_{r}}\right)}^{m}\right]$$

The definition of residual stress is the internal equilibrium stress that remains in a component after eliminating the external force or inhomogeneous temperature field. In order to abide by this principle, the stress relaxation procedure was considered by setting the workpiece as the thermal convection region when the cutting tool is removed. The film coefficient was 10 W/m^2^ °C and the sink temperature was 20 °C.

## 5. Results and Discussions

### 5.1. Validation of FE Model

**Figure 7:**
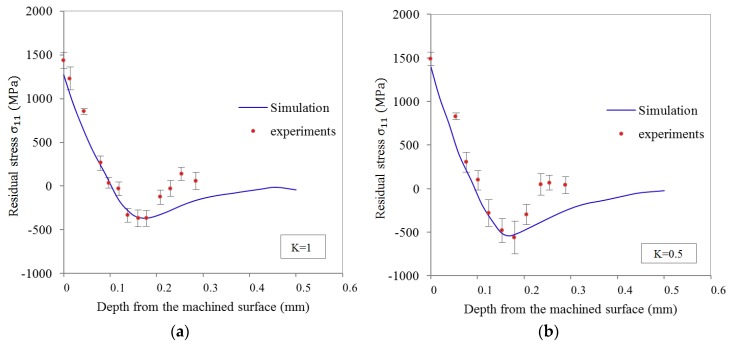
Comparison of residual stress by-depth profiles in cutting direction between simulation and experiments for different form factors *K*: (**a**) *K* = 1; (**b**) *K* = 0.5.

As shown in Figure 7, to validate the dependability of the numerical model, the simulated subsurface residual stresses profiles for unsymmetrical tools in cutting direction *σ*_11_ was compared with the experimental ones in the stable cutting stage. The residual stress profiles from experiments show more tensile and compressive stress than the simulated ones. The simplification of the material model and friction model in simulation, tool wear, and the inhomogeneity of workpiece material in experiments all cause error [15,24]. Although no agreement was achieved between the measured stress profiles and the predicted ones, which was also expected in advance, the FE model accurately predicted the residual stress contour shape and trends. Furthermore, the predicted values of maximum compressive residual stress and its depth were very close to the experimental ones. This indicates that the FE model can provide reliable and valid prediction of residual stress.

### 5.2. Formation Mechanism of Residual Stress

**Figure 8:**
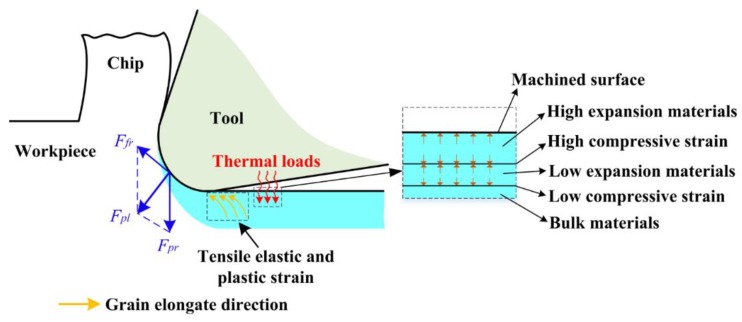
Schematic of the machined surface residual stress formation.

**Figure 9:**
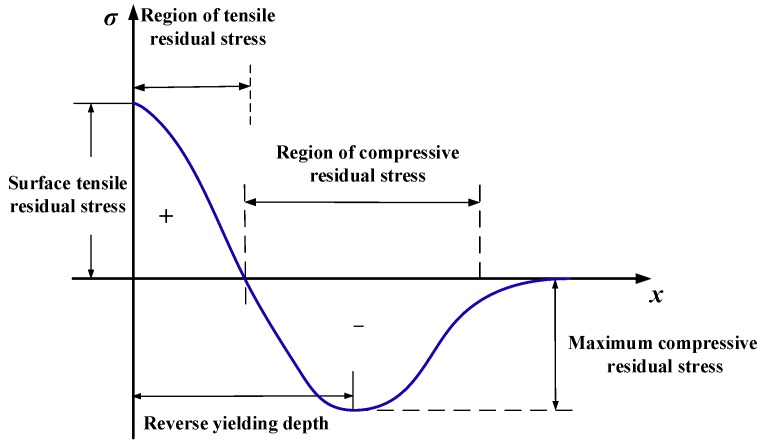
Representative features of the residual stress profile.

In order to better predict the residual stress states, a thorough understanding of the formation mechanism of machine-induced residual stress is needed. Figure 8 briefly illustrates the formation of machined surface residual stress. When cutting with a non-sharp edge, friction and ironing functions between the tool tip and material will be generated, which causes the ploughing effect. Due to the frictional function of ploughing force component *F_fr_* and the press function of ploughing force component *F_pr_*, a certain depth of material grain in the workpiece subsurface will be elongated in the cutting direction. Consequently, the tensile plastic strain and tensile elastic strain will be produced in the workpiece subsurface layer. After springing back, the release of the elastic strain tends to introduce compressive stress in the surface layer. It is believed that the magnitude of compressive stress is closely related to the depth of the ironed material, which will be demonstrated in a later section. Different degrees of thermal expansion deformation on the subsurface layer caused by cutting heat introduce a different degree of compressive strain in the surface layer. During cooling down, the retraction of the expansion is limited, which contributes to the formation of tensile stress. Since the formation of residual stresses is always subjected to the identical effect of thermomechanical phenomena, the residual stresses of machined surfaces conventionally have a homogeneous profile as shown in Figure 9. Tensile residual stresses on the surface layer of machined components were found to accelerate the expansion of cracks in some studies [3,25]. Inducing a higher depth or magnitude of compressive residual stresses in the surface or subsurface region will be favorable for fatigue life [26,27]. Furthermore, Guo et al. [27] suggested that the deep region of compressive residual stresses in the subsurface layer might be more beneficial to the fatigue life of bearing than shallower stresses region of greater magnitude. Based on the above research, the surface residual stress *σ_s_*, maximum compressive residual stress *σ_c_*, and its depth *h_r_* will be extracted as analytical indicators because of their influence on fatigue life. Several investigations [7,15,28] have proven that the generation of residual stress in feed direction *σ*_33_ mostly depends on *σ*_11_ and *σ*_33_ always shows the same changes with *σ*_11_. So the later analysis will focus on *σ*_11_ to discuss the effect of cutting tool microgeometry on residual stress in this research.

### 5.3. Effects of Cutting Edge Microgeometry on the Variation of Residual Stress

**Figure 10:**
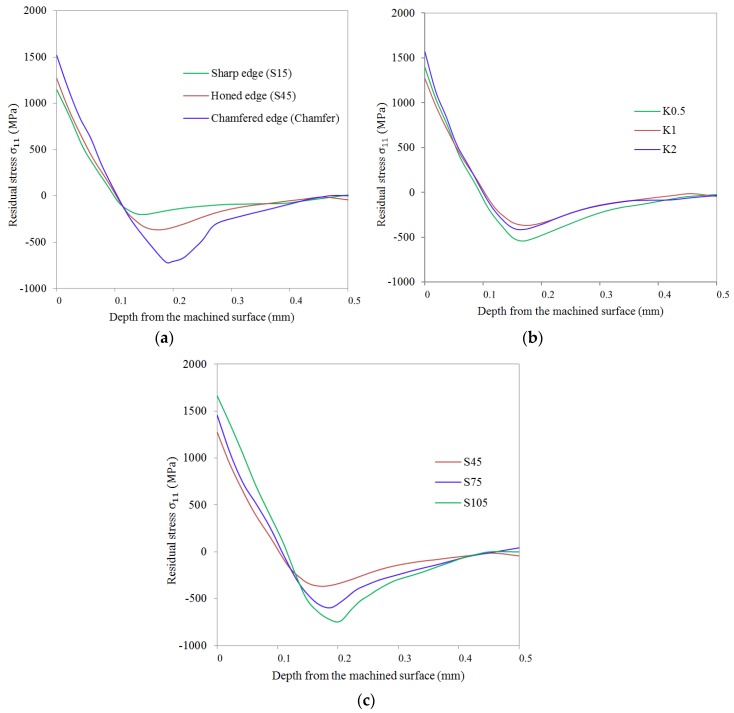
Residual stress profiles in cutting direction *σ*_11_ for (**a**) different cutting edge type, (**b**) honed cutting edge with different form-factor *K*, (**c**) symmetrical honed cutting edges with different average cutting edge radius $\bar{S}$.

Figure 10 showed the by-depth residual stress profiles in the cutting direction obtained from simulations. Compared to the honed cutting edge, it was clear that both the magnitude of surface tensile residual stress *σ_s_* and the maximum compressive residual stress *σ_c_* experience obvious growth when using a honed plus chamfer edge (see Figure 10a). The magnitude of the tensile and compressive residual stresses was smallest when using cutting tools with a sharp edge. The lowest tensile residual stress was induced when using the sharp cutting edge in simulations. However, using the sharp cutting edge is prone to cause deterioration of the surface quality due to rapid tool wear in the actual cutting process. Figure 10b,c showed that the form-factor *K* and the average cutting edge radius $\bar{S}$ have a predominant effect on the profiles of residual stress. For the unsymmetrical honed cutting edge of K0.5 and K2, the magnitude of residual stresses in both the tensile and compressive residual stress region is higher than K1. Furthermore, for K0.5 *σ_c_* showed a considerable increase while *σ_s_* showed an obvious increase for K2. It can be seen from Figure 10c that a larger $\bar{S}$ (or honed edge radius *r_β_*) produced a higher *σ_s_* and *σ_c_*. The *h_r_* goes slightly deeper into the workpiece for a cutting edge with a larger $\bar{S}$. The same phenomenon was observed in the experiments of turning AISI 316L [15]. However, in the research of orthogonal turning of AISI 52100, Hua et al. reported that *h_r_* remains almost unchanged for cutting tools with different *r_β_* [29]. This is mainly caused by the different thermal conductivity of the workpiece material. In the case of an asymmetrical cutting edge with $\bar{S}$ = 45 µm, changing its *K* to 0.5 or increasing $\bar{S}$ to 75 µm both intensify the *σ_s_* and *σ_c_* to a similar value (about 1400 MPa and −570 MPa, respectively). However, in the compressive stress depth region, when an asymmetrical cutting edge K0.5 is applied, a deeper region of compressive residual stress (about 315 µm) is shown compared to the 271 µm produced by symmetrical cutting edge S75. This has been proven more conducive to enhancing fatigue life [27].

### 5.4. Temperature Distribution and Tensile Residual Stress

**Figure 11:**
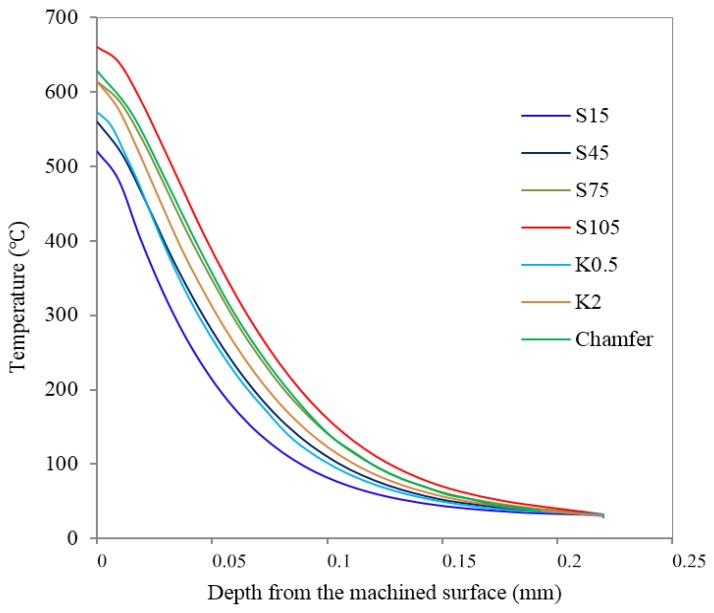
Temperature by-depth distribution with different cutting edge microgeometry.

**Figure 12:**
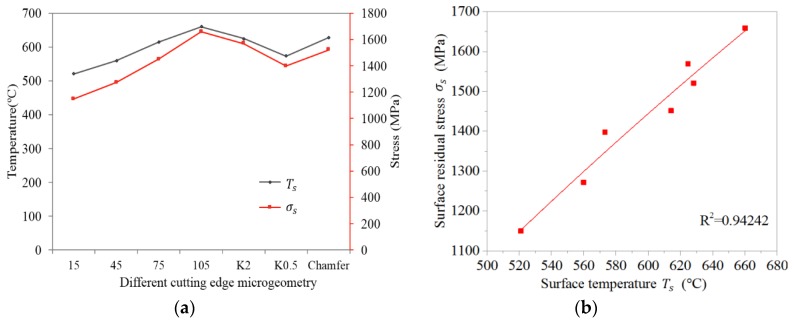
(**a**) The *T_s_* and *σ_s_* for different cutting edge microgeometry. (**b**) Changes of *σ_s_* with the increase of *T_s_*.

The values of cutting temperature varied with depth in the stable cutting period, as seen in Figure 11. It is easy to find that the temperature by-depth curves for all cutting edge microgeometries intersected at one point at a depth of 220 µm and approached the ambient temperature. This means the temperature influence depth for all cutting edge microgeometries was almost identical. In this case, a higher surface temperature is equivalent to a higher temperature gradient. It is seen from this figure that tools with larger $\bar{S}$ always produced a higher temperature, while the honed plus chamfer cutting edge provided the second largest cutting temperature. Moreover, for unsymmetrical honed edge K2 the cutting temperature is higher than K0.5 and K1. The contact length between the workpiece and the tool increased due to the larger $\bar{S}$ or chamfer, which led to an increase in the friction. As a result, a larger amount of cutting heat was generated. Meanwhile, the increase in contact length between the tool and workpiece also led to more cutting heat dissipating into the tools, which caused a decreasing trend of workpiece subsurface temperature. Obviously, the increased dissipated cutting heat is not sufficient to affect the increasing tendency of the subsurface temperature. The geometry of waterfall cutting edge K2 has a similar effect, increasing the tool/workpiece friction with honed plus chamfer edge, which explains the significant increase in *σ_s_* for K2 in Figure 10b. The increased temperature partially results from the heat released by the increased plastic deformation due to the increased ploughing depth, which will be introduced in the next section. Because of the significant influence of *σ_s_* on fatigue life, more attention was paid to the reason behind the change of *σ_s_* with different cutting edge microgeometries. The value of *σ_s_* is the result of thermal loads and mechanical loads. Figure 12a illustrates that the changes of surface temperature *T_s_* closely correspond to the changes of *σ_s_* with different cutting edge microgeometries. From Figure 12b, we can observe that *σ*_s_ varies almost linearly with *T_s_* and the determination coefficient reaches 0.942. Judging from this phenomenon, it can be concluded that the thermal load plays the dominant role in determining the surface residual stress *σ_s_*.

### 5.5. Stagnation Zone and Compressive Residual Stress

**Figure 13:**
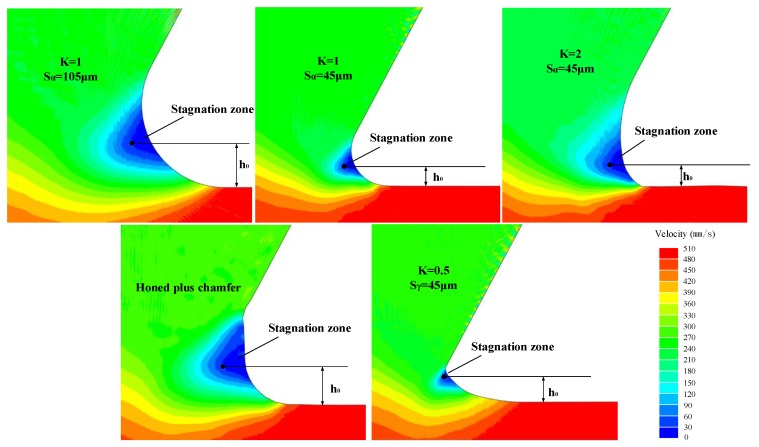
Stagnation zone in front of the cutting edge.

During cutting with non-sharp edges, there is a special area in front of the tool tip where the material flow rate is stationary relative to the cutting tool. This area is called the “stagnation zone” or “dead metal zone”. The formation of a stagnation zone is caused by the accumulation and obstruction of material underneath the tool tip. Figure 13 demonstrates that the characteristics of the stagnation zone depend on the microgeometry of the cutting edge. The distribution of workpiece material velocity near the tool tip with different cutting edge microgeometries during the stable cutting period is shown in Figure 13, where the stagnation zone is distinguished by a dark blue color. As reported in many studies [15,30], the size of the stagnation zone increased when introducing a chamfer or using a cutting edge with a bigger $\bar{S}$ (or *r_β_*). Bassett et al. [11] also demonstrated that when using a honed cutting edge with *K* > 1, a stagnation zone can be observed, while for a cutting edge with *K* < 1 the stagnation zone cannot be recognized. The stagnation zone acts as an extension of the cutting edge and plays a protective role to the cutting edge during machining.

**Figure 14:**
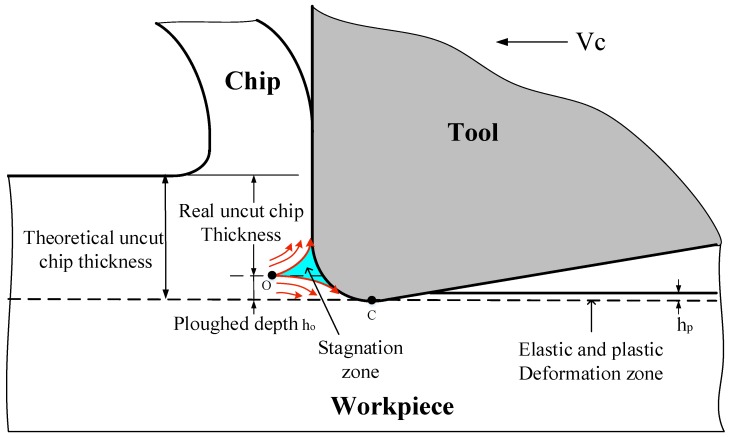
Material flow and phenomenon of uncut chip thickness reducing in ploughing effect.

**Figure 15:**
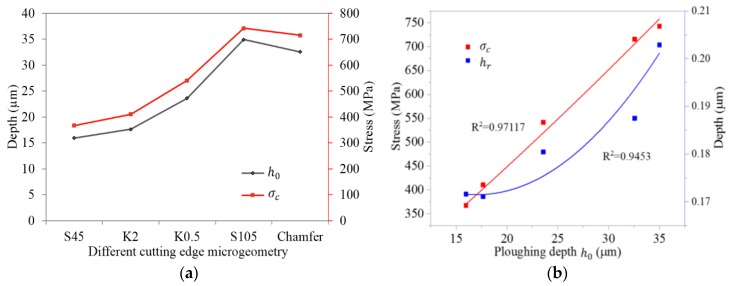
(**a**) *h_o_* and *σ_c_* for different cutting edge microgeometry. (**b**) Changes of *h_r_* and *σ_c_* with different *h_o_*.

Since the stagnation zone affects the ploughing effect, which has a dramatic influence on the machined surface residual stress, more attention should be paid to understanding of it. Figure 14 briefly illustrates the phenomenon of uncut chip thickness reducing in ploughing effect. The stagnation zone appears as a triangle with one vertex O acting as the separation point of the workpiece material. In the range of theoretical uncut chip thickness, the material below point O will flow downwards and be ironed by the cutting edge. Meanwhile, strong elastic and plastic tensile strain has been generated. This ironed material depth is the ploughing depth *h_o_*. As shown in Figure 15a, when K0.5 and K2 were applied, *h_o_* increases compared to K1, while for K0.5 *h_o_* shows a more significant increase. Increasing $\bar{S}$ or introducing a chamfer results in increasing the value of *h_o_*. The increase of *h_o_* leads to more material below point O being ironed downwards and further results in the generation of stronger plastic deformation on the machined surface layer. Since mechanical plastic deformation tends to induce compressive residual stress, the increasing trend observed in the values of *σ_c_* as *h_o_* increased, as shown in Figure 15b, can be explained. Furthermore, it is noteworthy that the maximum compressive residual stress *σ_c_* has an almost proportional relationship with *h_o_*, which convincingly demonstrates that the mechanical loads play a dominant role in the formation of residual stress at the *h_r_*. The *h_r_* shows an increasing trend on the whole with the increasing of *h_o_* in Figure 15b. This is because the higher ploughing depth leads to deeper ranging plastic deformation and therefore higher *h_r_*.

It should be noted that the profile of residual stress is not a direct consequence of either the thermal load or the mechanical load, but a complex result of both of them. Increasing $\bar{S}$ means the tool tip becomes bigger, which in turn contributes to two conflicting phenomena. On the one hand, the deeper ranging ironed workpiece material (as shown in Figure 13) leads to a higher compressive residual stress. On the other hand, the larger friction between the tool tip and workpiece causes more heat generation and therefore higher tensile residual stress (as shown in Figure 11). No cutting temperature gradient is significantly deeper than 220 μm below the surface. The thermal effect is only remarkable in the near-surface layer and declines quickly with depth. With reference to Figure 12 and Figure 15, we can conclude that the tensile stress caused by thermal loads has a leading effect on residual stress profiles above *h_r_* while the compressive stress caused by mechanical loads plays a major role below the *h_r_*. When *h_o_* increases, the elasto-plastic deformation on the machined surface will also increase and more heat will be released. This is another important cause of the increase in cutting temperature. Therefore, *T_s_* tends to be higher for K0.5 compared to for K1.

## 6. Conclusions

An FE model for predicting the effect of microgeometry on residual stress profile of machined surface is investigated. According to the numerical results, the conclusions can be listed as follows.

1. With the increase in average cutting edge radius $\bar{S}$, the values of the surface tensile residual stress *σ_s_* and the maximum compressive residual stress *σ_c_* present an increasing trend. Using the honed plus chamfer cutting edge plays a similar role with using a cutting edge with a larger average cutting edge radius $\bar{S}$, increasing the magnitude of residual stress.

2. The increasing surface tensile residual stress *σ_s_* is attributed to the higher temperature gradient in the workpiece subsurface layer for a cutting edge with a bigger average cutting edge radius $\bar{S}$ or honed plus chamfer cutting edge, as more heat is generated due to the increasing frictional contact length between the cutting edge and the workpiece. Another reason for the temperature increasing is the deeper plastic deformation caused by an increase of ploughing depth *h_o_*, which releases more heat.

3. The phenomenon of a stagnation zone is analyzed as a limitation of workpiece material diversion to predict ploughing depth *h_o_*. The increase of *h_o_* means more subsurface material is ironed on the deformation zone, which provides a specific explanation for the intensification of maximum compressive residual stress *σ_c_*.

4. When a cutting edge with form-factor *K* = 2 is used, the friction between the tool tip and workpiece increased, which consequently contributes to the increase in cutting temperature and *σ_s_*. For a cutting edge with *K* = 0.5, *h_o_* increased and a higher *σ_c_* can be achieved.

## Figures and Tables

**Table 1:** Parameters of electrolytic polishing.

**Electrolyte Composition**	CH_3_OH/HNO_3_	200 mL/100 mL
**Electrolytic Parameters**	Voltage Current density	10 V 0.5~0.7 A/cm^2^
**Environment**	Room temperature (20 °C)	

**Table 2:** Johnson–Cook constitutive model parameters of Inconel 718 [23].

*A* (MPa)	*B* (MPa)	*C*	*n*	*m*
450	1700	0.017	0.65	1.3

**Table 3:** The physical and thermomechanical properties of the tool and the workpiece material.

Properties	Density	Young’s Modulus	Poisson’s Ratio	Thermal Expansion	Conductivity	Specific Heat
Tool	14,800 Kg/m^3^	640 GPa	0.22	4.5 μm/mK	50.24 W/mK	220 J/kgK
Workpiece	8250 Kg/m^3^	214.580 GPa	0.305	14.8 μm/mK	17.8 W/mK	526.3 J/kgK
